# Anti-Tick Microbiota Vaccine Impacts *Ixodes ricinus* Performance during Feeding

**DOI:** 10.3390/vaccines8040702

**Published:** 2020-11-21

**Authors:** Lourdes Mateos-Hernández, Dasiel Obregón, Jennifer Maye, Jeremie Borneres, Nicolas Versille, José de la Fuente, Agustín Estrada-Peña, Adnan Hodžić, Ladislav Šimo, Alejandro Cabezas-Cruz

**Affiliations:** 1 UMR BIPAR, INRAE, ANSES, Ecole Nationale Vétérinaire d’Alfort, Université Paris-Est, Marie Curie, 94706 Maisons-Alfort, France; ladislav.simo@vet-alfort.fr; 2 School of Environmental Sciences, University of Guelph, Guelph, ON N1G 2W1, Canada; dasielogv@gmail.com; 3 Center for Nuclear Energy in Agriculture, University of São Paulo, Piracicaba 13400-970, Brazil; 4 SEPPIC Paris La Défense, 92250 La Garenne Colombes, France; jennifer.maye@airliquide.com (J.M.); jeremie.borneres@airliquide.com (J.B.); Nicolas.VERSILLE@airliquide.com (N.V.); 5 SaBio, Instituto de Investigación en Recursos Cinegéticos (IREC-CSIC-UCLM-JCCM), 13005 Ciudad Real, Spain; jose_delafuente@yahoo.com; 6 Department of Veterinary Pathobiology, Center for Veterinary Health Sciences, Oklahoma State University, Stillwater, OK 74078, USA; 7 Faculty of Veterinary Medicine, University of Zaragoza, 50009 Zaragoza, Spain; aestrada@unizar.es; 8 Institute of Parasitology, Department of Pathobiology, University of Veterinary Medicine Vienna, Vienna 1210, Austria; Adnan.Hodzic@vetmeduni.ac.at

**Keywords:** anti-tick microbiota vaccines, tick control, α-Gal

## Abstract

The tick microbiota is a highly complex ensemble of interacting microorganisms. Keystone taxa, with a central role in the microbial networks, support the stability and fitness of the microbial communities. The keystoneness of taxa in the tick microbiota can be inferred from microbial co-occurrence networks. Microbes with high centrality indexes are highly connected with other taxa of the microbiota and are expected to provide important resources to the microbial community and/or the tick. We reasoned that disturbance of vector microbiota by removal of ubiquitous and abundant keystone bacteria may disrupt the tick-microbiota homeostasis causing harm to the tick host. These observations and reasoning prompted us to test the hypothesis that antibodies targeting keystone bacteria may harm the ticks during feeding on immunized hosts. To this aim, in silico analyses were conducted to identify keystone bacteria in the microbiota of *Ixodes* nymphs. The family Enterobacteriaceae was among the top keystone taxa identified in *Ixodes* microbiota. Immunization of α-1,3-galactosyltransferase-deficient-C57BL/6 (α1,3GT KO) mice with a live vaccine containing the Enterobacteriaceae bacterium *Escherichia coli* strain BL21 revealed that the production of anti-*E. coli* and anti-α-Gal IgM and IgG was associated with high mortality of *I. ricinus* nymphs during feeding. However, this effect was absent in two different strains of wild type mice, BALB/c and C57BL/6. This result concurred with a wide distribution of α-1,3-galactosyltransferase genes, and possibly α-Gal, in Enterobacteriaceae and other bacteria of tick microbiota. Interestingly, the weight of *I. ricinus* nymphs that fed on *E. coli*-immunized C57BL/6 was significantly higher than the weight of ticks that fed on C57BL/6 immunized with a mock vaccine. Our results suggest that anti-tick microbiota vaccines are a promising tool for the experimental manipulation of vector microbiota, and potentially the control of ticks and tick-borne pathogens.

## 1. Introduction

Non-pathogenic microbes associated with ticks impact vector physiology and survival [1]. Some endosymbionts, such as *Francisella* sp., contribute to the synthesis of vitamin B, a nutritional component essential for ticks and absent in the blood meal. The nutritional complementation by *Francisella* sp. is fundamental for tick development and survival [1]. Beyond endosymbiotic bacteria, other genera such as *Pseudomonas*, *Sphingobacterium*, *Acinetobacter*, *Enterobacter*, and *Stenotrophomonas* are common colonizers of the midgut in hard ticks [2]. Vitamin B synthesis genes are not restricted to *Francisella* [1] or *Coxiella* [3], but are widely distributed in some genera of tick microbiota [4]. This suggests that nutritional complementation in ticks can be an attribute not only of symbionts, but also of tick microbiota bacteria [4]. In addition, the diversity of genes and metabolic pathways encoded in the bacterial genomes of the tick microbiome suggests that the contribution of bacteria to tick physiology and survival could go beyond vitamin B supplementation [4,5]. A functional complementation between tick and gut microbiota genomes is expected, considering the strong phylosymbiotic signal of microbial communities associated to Ixodid ticks [6]. However, the theoretical prediction of tick-microbiota phylosymbiosis has not been empirically tested [6]. To fill this gap, it is necessary to develop tools to assess the role of specific microbiota bacteria in tick development, physiology and survival. Microbial communities harbor keystone taxa which are highly connected and have a great explanatory power of the community structure and functioning irrespective of their abundance [7]. Keystone taxa drive community composition and function and can be identified using co-occurrence networks [7,8,9]. Due to their central role in the microbial networks, removal or addition of keystone taxa may be associated with major shifts in the whole community structure.

The synthesis of the glycan Galα1-3Galβ1-4GlcNAc-R (α-Gal) by the enzyme α-1,3-galactosyltransferase occurs in bacteria [10,11,12,13], fungi [14,15,16], and noncatarrhine mammals [17], but prokaryotic and eukaryotic α-1,3-galactosyltransferase genes and proteins share little structural homology [18,19,20]. Humans, old world monkeys and apes evolved with the inability to synthesize α-Gal, which resulted in the capacity to produce anti-α-Gal IgM/IgG antibodies with a protective activity against pathogenic viruses (e.g., HIV), bacteria (e.g., *Mycobacterium*) and parasites (e.g., *Plasmodium*), containing this modification on membrane proteins [21,22,23]. The natural IgM/IgG antibodies against α-Gal are produced in response to gut microbiota bacteria having this modification [21,24]. Several bacteria of the family Enterobacteriaceae such as *Klebsiella pneumonia* [10], *Escherichia coli* [11,12] and *Salmonella* spp. [13,24] are known to express α1,3-galactosyltransferase genes, and to synthetize α-Gal, in the human gut microbiome [24]. Similar to humans, nonmammalian vertebrates such as fish, amphibians, reptiles and birds do not express the α-Gal epitope [17,25,26], which makes them able to produce anti-α-Gal antibodies in response to gut colonization with bacteria expressing α-Gal. Tick saliva contains glycoproteins with α-Gal modifications that can induce in some individuals the production of high levels of anti-α-Gal IgE associated with allergic reactions [27,28,29,30]. It has been proposed that the presence of α-Gal in ticks may be a mechanism of molecular mimicry of the vector to escape the immunity of the vertebrate host producing endogenous α-Gal and incapable of producing high levels of natural anti-α-Gal antibodies [19,31]. Currently, three hypotheses explain that the origin of α-Gal in ticks could be due to: (i) residual mammalian glycoproteins or glycolipids from a previous blood meal of a nonprimate mammal, (ii) endogenous α-Gal synthesis by ticks, and (iii) pathogenic or non-pathogenic bacteria (symbionts or microbiota) present in the ticks and able to produce α-Gal. Evidence supporting hypotheses (i) and (ii) have been published [29], while the hypothesis in (iii) remains to be tested.

In this work, we used network analysis to identify keystone taxa in tick gut microbiota. A live bacteria vaccine was then used to target Enterobacteriaceae bacteria, one of the identified keystone taxon. Using a functional metagenomic approach, we predicted the presence of α1,3-galactosyltransferase genes in Enterobacteriaceae and other bacteria of the tick gut microbiome. Our results showed for the first time that immunization with defined members of tick gut microbiota can be used to modulate tick physiology and microbiota composition. In addition, the results suggest that tick microbiota may be a source of α-Gal with unknown relevance for tick-induced allergy and that anti-α-Gal immunity may play a protective role against tick infestation. We conclude that host immunization with selected vector microbiota bacteria is a promising tool for the experimental manipulation of vector gut microbiota, and potentially vector and vector-borne pathogen control.

## 2. Results

### 2.1. Enterobacteriaceae Is a Keystone Bacterial Family in Ixodes Gut Microbiota

**Figure 1:**
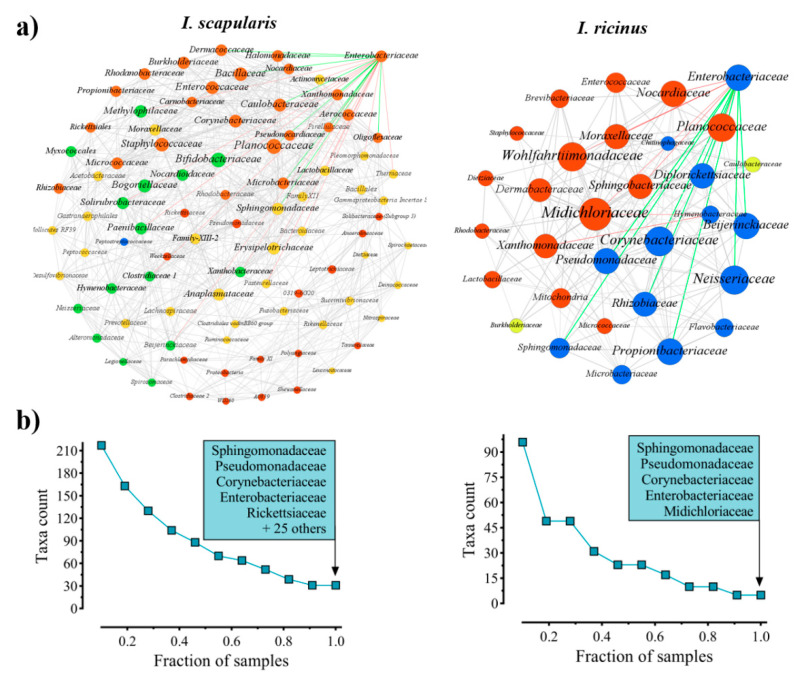
Interactions and ubiquitous bacterial families on the gut microbiota of *I. scapularis* and *I. ricinus* nymphs. (**a**) Co-occurrence bacterial networks obtained from 16S sequences. Nodes correspond to taxa (family level), and connecting edges indicate significant (*p* < 0.01) and strong positive or negative correlations (SparCC > 0.7 or < −0.7). Only nodes with at least one significant correlation are represented. Node colors are based on modularity class metric, hence the same color mean modules of co-occurring taxa. The size of the nodes is proportional to the eigencentrality of each taxon. Enterobacteriaceae is highlighted (top right), and the connecting edges differentiated in positive (green) and negatives (red). (**b**) Identification of the ubiquitous families across the samples in each data set. Four families were found in all samples (*n* = 20) from both *I. scapularis* and *I. ricinus* nymphs.

In the first step of this work, we used a multifactor approach for the screening of potential keystone taxa in the gut microbiota of *Ixodes* ticks, based on: (i) eigencentrality, (ii) ubiquitousness, and (iii) the combination of relative abundance and eigencentrality. Two previously published datasets [32,33], from 16S rRNA (16S) sequences of *Ixodes* gut microbiota were used to identify keystone taxa. One dataset from *Ixodes scapularis* nymphs reared in laboratory conditions [32], and the other from free-living *Ixodes ricinus* nymphs collected in the Swiss Alps at different altitudes [33]. Bacteria co-occurrence networks were used to quantify and rank the centrality of bacterial families present in the gut microbiota of *I. scapularis* and *I. ricinus* nymphs. The selection of bacteria was based on eigencentrality, which is a measure of the influence of a node in a network [34]. Several bacterial families resulted central in the co-occurrence networks from both *I. scapularis* and *I. ricinus* microbiota (Figure 1a). The ubiquitousness of the taxa was addressed by identifying the microbial families present across all the samples of *I. scapularis* and *I. ricinus* nymphs. The analysis revealed greater taxon richness in the *I. scapularis* microbiota compared with *I. ricinus*. Thirty families were identified as the core microbiota of *I. scapularis*, while in *I. ricinus* only five families were present in all the samples (Figure 1b). Only four families were found in all samples from both datasets: Enterobacteriaceae, Corynebacteriaceae, Pseudomonadaceae and Sphingomonadaceae.

**Figure 2:**
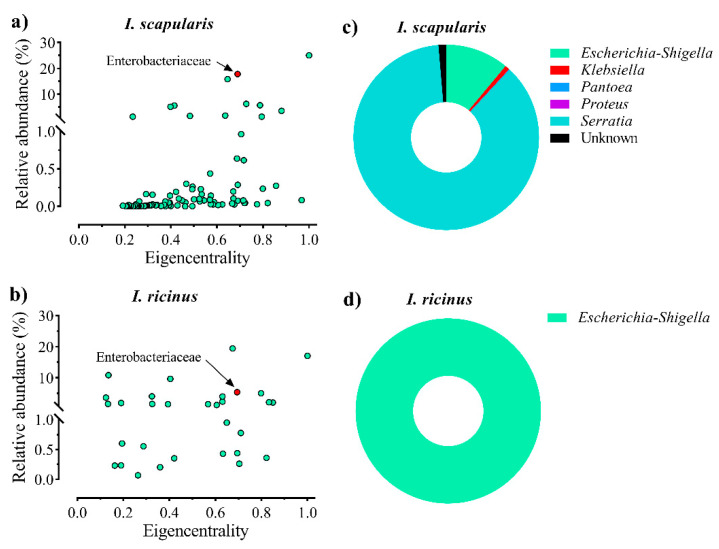
Eigencentrality and relative abundance of bacterial families in *I. scapularis* and *I. ricinus* microbiota. Relative abundance of all bacterial families was plotted as a function of their eigencentrality in the co-occurrence networks of (**a**) *I. scapularis* and (**b**) *I. ricinus* microbiota. Only families with at least one significant correlation with other nodes in the networks are displayed. Circles represent bacterial families and Enterobacteriaceae was highlighted (red color). The taxonomic composition (%) of Enterobacteriaceae according to bacterial genera are represented in donut chart for both (**c**) *I. scapularis* and (**d**) *I. ricinus* microbiota.

As a third screening criterion, we used the combination of relative abundance and eigencentrality. The list of bacterial families identified in *I. scapularis* and *I. ricinus*, including relative abundance and eigencentrality values, is available as Supplementary Table S1. In both *I. scapularis* (Figure 2a) and *I. ricinus* (Figure 2b), Enterobacteriaceae was among the bacterial families with the highest relative abundance and eigencentrality. Among the four ubiquitous families in both *Ixodes* species (Figure 1b), Enterobacteriaceae and Corynebacteriaceae had the highest values of eigencentrality and relative abundance (Table 1). Based on the ubiquitousness, high eigencentrality, and the combination of high relative abundance and eigencentrality in both *Ixodes* species, Enterobacteriaceae and Corynebacteriaceae were selected as keystone taxa. However, in this study, only Enterobacteriaceae was used in downstream experiments, as a proof of concept. Within the family Enterobacteriaceae, bacteria of the genus *Escherichia*-*Shigella* were the second most represented taxa in *I. scapularis* (Figure 2c) and the only taxa represented in *I. ricinus* (Figure 2d).

### 2.2. α1,3-Galactosyltransferase Genes Are Broadly Distributed in the Tick Gut Microbiome

**Figure 3:**
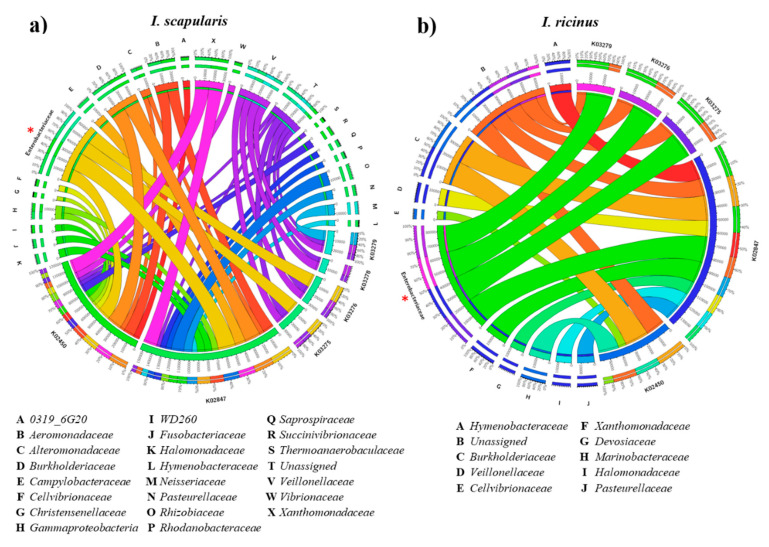
Metagenome contribution to α1,3-galactosyltransferase genes in the gut microbiota of (**a**) *I. scapularis*, and (**b**) *I. ricinus* nymphs. The position of Enterobacteriaceae was highlighted (red *). KEGG orthology and taxonomic contribution was predicted using PICRUSt2. Chord diagrams show the linkage between classified and unclassified (unassigned) taxa and α1,3-galactosyltransferase genes. The arcs indicate connections, represented proportionally by the size of each arc. Node segments along a circle represent taxa or functional genes, the node size indicates the abundance (measured as feature count) of contributing taxa and genes. Chord diagrams were made with the visualization tool “Circos” [37].

Bacteria of the family Enterobacteriaceae are known to express α1,3-galactosyltransferase genes and to synthetize the α-Gal glycan in the human gut microbiome [10,11,12,13,24,35]. However, the presence and distribution of these genes in the tick microbiome remain unknown. Here, we used the functional metagenomic inference approach PICRUSt2 [36] to assess the distribution of α1,3-galactosyltransferase bacterial genes in *Ixodes* gut microbiota. Several α1,3-galactosyltransferase genes including *gspA*-general secretion pathway protein A (K02450), *waaL*, *rfaL*-O-antigen ligase [EC:2.4.1.-] (K02847), *waaO*, *rfaI*-UDP-glucose: (glucosyl) LPS alpha-1,3-glucosyltransferase [EC:2.4.1.-] (K03275), *waaJ*, *rfaJ*; UDP-glucose: (galactosyl) LPS alpha-1,2-glucosyltransferase [EC:2.4.1.58] (K03279) and *waaR*, *waaT*, *rfaJ*- UDP-glucose/galactose:(glucosyl)LPS alpha-1,2-glucosyl/galactosyltransferase [EC:2.4.1.-] (K03276) were identified in the microbiome of *I. scapularis* and *I. ricinus*. The gene *waaI*, *rfaI*-UDP-D-galactose: (glucosyl) LPS alpha-1,3-D-galactosyltransferase [EC:2.4.1.44] (K03278) was identified only in *I. scapularis* (Figure 3). The PICRUSt2 pipeline also allowed tracing the contribution of each 16S amplicon sequence variant (ASV) to the predicted genes. In *I. scapularis*, the α-1,3-galactosyltransferase genes were traced to 22 classified bacterial families, one ASV from the phylum Gammaproteobacteria, unclassified at the family level, and other unassigned ASV (Figure 3a). Among these genes, the KO2847 and KO2450 were the most abundant on the functional microbiome of *I. scapularis*, and the largest contribution came from Enterobacteriaceae bacteria metagenomes, followed by Burkholderiaceae, and the set of unassigned ASVs. In the *I. ricinus* microbiome, the five genes were traced to 11 classified bacterial families and, as in *I. scapularis*, the genes KO2847 and KO2450 were the most abundant on the functional microbiome of *I. ricinus* (Figure 3b).

To validate the results generated by PICRUSt2, we tested midguts of 25 laboratory-reared *I. ricinus* female ticks for the presence of the selected bacterial α1,3-galactosyltransferase genes. Overall, two out of five pools tested (midguts of five ticks per pool) showed positive results in PCRs and sequence analyses revealed 100% identity to the corresponding genes expressed by different *E. coli* and *Shigella* sp. strains. PCR amplification of waaR gene revealed a band of the expected size (685 bp), but we failed to sequence the amplicon. The representative nucleotide sequences of the bacterial α1,3-galactosyltransferase genes were deposited in the GenBank database and are available under the following accession numbers: MW222477 (*gspA*—general secretion pathway protein A), MW222479 (*waaL*, *rfaL*-O-antigen ligase), MW222480 (*waaO*, *rfaI*-UDP-glucose:(glucosyl)LPS alpha-1,3-glucosyltransferase), and MW222478 (*waaJ*, *rfaJ*-UDP-glucose:(galactosyl)LPS alpha-1,2-glucosyltransferase).

### 2.3. Anti-α-Gal Immunity and Host Genetic Background Influence the Impact of Live E. coli Vaccination on Tick Performance

**Figure 4:**
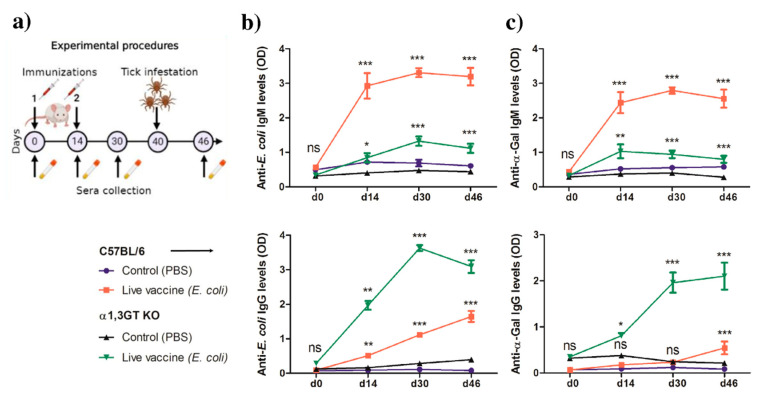
Antibody response of C57BL/6 and α1,3GT KO mice after immunization with a live *E. coli* vaccine. (**a**) Mice were immunized twice and then infested with *I. ricinus* nymphs. The levels of IgM and IgG to *E. coli* proteins (**b**) and α-Gal (**c**) were quantified in sera by ELISA. Results shown are means and standard error values. Results were compared by two-way ANOVA with Bonferroni test applied for comparisons between control and immunized mice within each strain C57BL/6 or α1,3GT KO (* *p* < 0.05, ** *p* < 0.001, *** *p* < 0.0001, ns—not significant; 1 experiment for each mice strain, *n* = 10 α1,3GT KO mice, *n* = 10 C57BL/6 mice and three technical replicates per sample).

The initial hypothesis of this study was that targeting keystone bacteria within the tick microbiota with host antibodies could impact tick performance. The discovery of Enterobacteriaceae as a keystone taxon in *Ixodes* microbiota, and the high distribution of α1,3-galactosyltransferase genes in Enterobacteriaceae and other tick microbiota bacteria, prompted us to test the impact of anti-α-Gal immunity on tick performance after immunization with Enterobacteriaceae bacteria. To this aim, we used the α-1,3-galactosyltransferase-deficient-C57BL/6 (α1,3GT KO) mice, the only available nonprimate mammals that can produce natural anti-α-Gal IgM and IgG in titers similar to those in humans. Wild type C57BL/6 mice having the same genetic background as α1,3GT KO mice, but expressing a functional copy of the *ggta1* gene, were used as controls. Mice were immunized with a live vaccine containing the Enterobacteriaceae bacteria *E. coli* strain BL21. Sera samples were collected during the experiment (Figure 4a). Immunization with *E. coli* induced the production of IgM and IgG to *E. coli* proteins in both α1,3GT KO and C57BL/6 mice (Figure 4b). The levels of anti-α-Gal IgM increased after immunization in both mice strains when compared with the control animals of the same strain (Figure 4c). However, C57BL/6 mice failed to developed anti-α-Gal IgG even sixteen days (d30) after the second immunization, while this antibody increased significantly in α1,3GT KO mice after the first immunization (d14). A slight, but significant, increase in anti-α-Gal IgG was recorded in C57BL/6 mice on d46 (Figure 4c).

**Figure 5:**
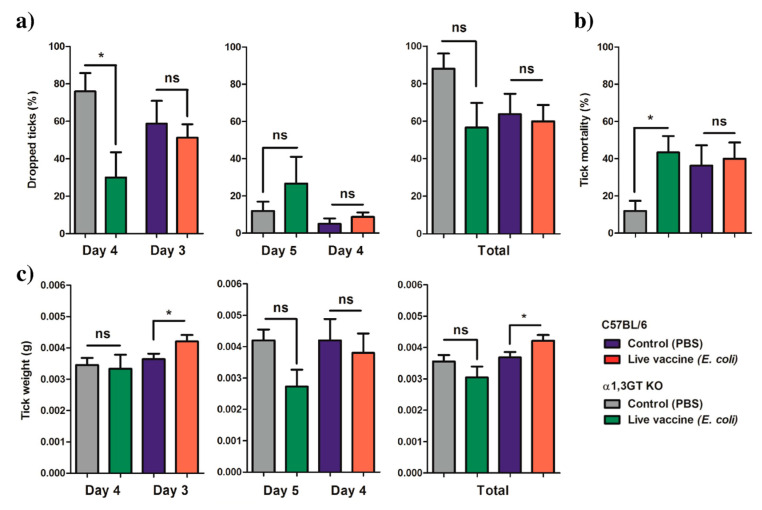
Performance of *I. ricinus* nymphs feeding on α1,3GT KO and C57BL/6 mice immunized with live *E. coli*. (**a**) The percentage of ticks that dropped on day 3, 4 or 5, and the total, was calculated and compared between groups. (**b**) The percentage of dead ticks was calculated and tick mortality (%) compared between groups. (**c**) At the end of feeding, the weight of individual ticks was measured and compared between groups. Means and standard deviation values are displayed. Results were compared within each mouse strain by Mann−Whitney U test. (* *p* < 0.05, ns—not significant; 1 experiment for each mouse strain, *n* = 12 α1,3GT KO mice (*n* = 5 ticks per mouse), *n* = 10 C57BL/6 mice (*n* = 20 ticks per mouse)).

Mice developed a white vaccine deposit located at the injection point due to the nature of the adjuvant. However, no mortality was associated with *E. coli* immunization and no sign of pain was observed after the vaccination. We hypothesized that the water-in-oil emulsion of the vaccine protected the mice from a sudden discharge of the bacteria, avoiding sepsis and a septic shock. Following the immunization protocol, each mouse was infested with 5 (α1,3GT KO) or 20 (C57BL/6) *I. ricinus* nymphs. Time to complete feeding, weight of engorged ticks and tick mortality were recorded and compared between immunized and control groups of the same mice strain (Figure 5). The ticks feeding on *E. coli*-immunized α1,3GT KO mice started dropping at d4, one day after the ticks that were feeding on the *E. coli*-immunized C57BL/6 mice (Figure 5a). More than 70% of *I. ricinus* nymphs that fed on control α1,3GT KO mice dropped on day 4, while less than 40% of the ticks that fed on the *E. coli*-immunized mice dropped on day 4 (Figure 5a). There were no significant differences in the proportion of ticks that dropped from *E. coli*-immunized or control C57BL/6 mice on d3, d4 or α1,3GT KO on d5 or in the total amount of ticks that dropped from each mouse regardless of the strain (Figure 5a). Immunization with *E. coli* did not affect the mortality of ticks that fed on C57BL/6 mice, but produced a significant increase in the mortality of ticks that fed on α1,3GT KO (Figure 5b). The weight of ticks that dropped on d4, d5 (α1,3GT KO) and d4 (C57BL/6) was not affected by *E. coli* immunization (Figure 5c). However, the weight of ticks that dropped from *E. coli*-immunized C57BL/6 on d3 was significantly higher than the ticks that fed on the control group. The weight of all ticks from *E. coli*-immunized C57BL/6 was also significantly higher than the control (Figure 5c).

**Figure 6:**
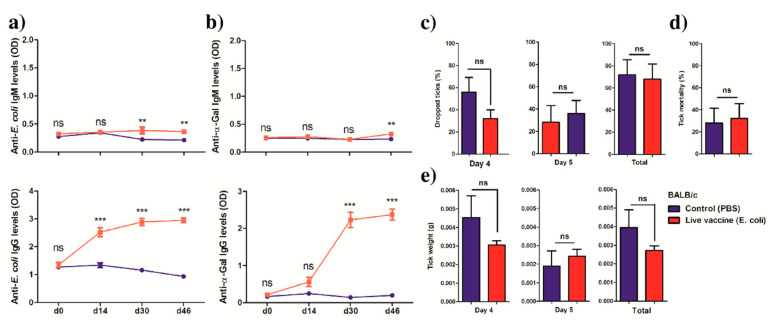
Antibody response of BALB/c mice and tick performance after *E. coli* immunization. The levels of IgM and IgG levels to (**a**) *E. coli* proteins and (**b**) α-Gal were quantified in sera by ELISA. (**c**) The percentage of ticks that dropped on days 4 or 5, and the total, were calculated and compared between groups. (**d**) The percentage of dead ticks was calculated and tick mortality (%) compared between groups. (**e**) At the end of the feeding, the weight of individual ticks was measured and compared between groups. Means, standard error (**a**,**b**) and standard deviation (**c**–**e**) values are shown. Results were compared by two-way ANOVA with Bonferroni multiple comparison test applied for individual comparisons (ELISA) and Mann−Whitney U test (tick performance). (** *p* < 0.001, *** *p* < 0.0001; ns—not significant; 1 experiment, *n* = 10 mice (*n* = 5 ticks per mouse) and three technical replicates per sample (ELISA).

To further test the role of the anti-microbiota vaccine, another mouse strain, BALB/c, was immunized and infested with ticks following the experimental procedures presented in Figure 4a. BALB/c mice express a functional copy of the *ggta1* gene and are not capable of producing natural anti-α-Gal antibodies, as C57BL/6. Immunization with *E. coli* induced the production of IgG to *E. coli* proteins (Figure 6a) and α-Gal (Figure 6b) in BALB/c mice. However, these mice failed to produced anti-*E. coli* (Figure 6a) and anti-α-Gal IgM in response to *E. coli* immunization (Figure 6b). None of the parameters measured on the ticks, i.e., time to complete feeding (Figure 6c), tick mortality (Figure 6d) and weight of engorged ticks (Figure 6e), was significantly affected after *E. coli* vaccination in BALB/c mice.

### 2.4. Live E. coli Vaccination in α1,3GT KO Mice Is Associated with Decreased Relative Abundance of Enterobacteriaceae in I. ricinus

**Figure 7:**
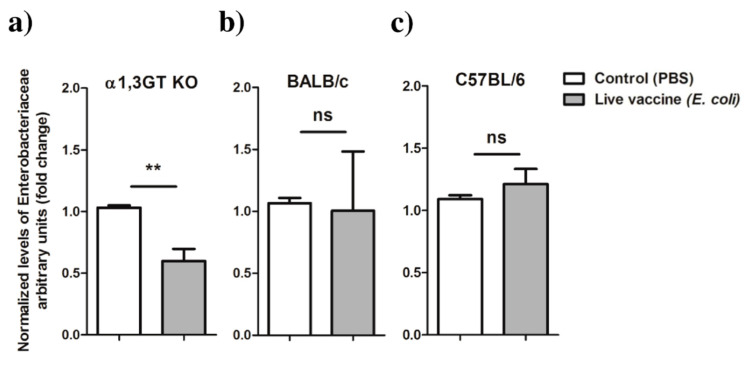
Relative abundance of Enterobacteriaceae in *I. ricinus* microbiota after feeding on *E. coli*-immunized mice. The relative abundance of Enterobacteriaceae was measured in adult ticks that in the previous stage (i.e., nymphs) had fed on (**a**) α1,3GT KO or (**b**) BALB/c and in engorged nymphs that fed on (**c**) C57BL/6 mice. Relative bacterial DNA levels were measured using Enterobacteriaceae-specific 16S qPCR normalizing against tick *rsp4* with the 2^−ΔΔCt^ ratio method. Results are relative to 16S in the control group. Means and standard deviation values are displayed. Results were compared within each mouse strain by Mann−Whitney U test. (** *p* < 0.001, ns—not significant; *n* = 11 pools of ticks that fed α1,3GT KO mice, *n* = 9 pools of ticks that fed BALB/c, *n* = 18 pools of ticks that fed C57BL/6 mice).

We then asked whether vaccination with live *E. coli* was associated with changes in the abundance of Enterobacteriaceae bacteria in tick microbiota. Nymphs that fed on α1,3GT KO and BALB/c mice immunized with *E. coli* or mock vaccine, were left to molt to adults. After molting, ticks were pooled, the DNA extracted and the normalized levels of Enterobacteriaceae 16S were measured by qPCR. Total DNA was also extracted from engorged nymphs that fed on C57BL/6 mice. The results showed a significant decrease in the relative abundance of Enterobacteriaceae in adult ticks that had fed on *E. coli*-immunized α1,3GT KO, compared to the control (Figure 7a). However, no significant changes in Enterobacteriaceae 16S abundance was found in the ticks that fed on either of the *E. coli*-immunized wild type strains, regardless of the developmental stage, unfed adults (BALB/c, Figure 7b) or fed nymphs (C57BL/6, Figure 7c).

## 3. Discussion

Ticks harbor a high diversity of bacterial species, likely as part of their evolutionary strategy to cope with their complex lifecycle and metabolic deficiencies [38]. Evidence suggests that some bacteria have key roles in the structure, organization and functioning of tick microbiota, since they constitute a reduced taxonomic core of the tick gut microbiota that remains stable despite biological disturbance [5]. Most importantly, the ubiquitousness of these taxa is likely associated to benefits they provide to the microbial community or the host [4,5,6]. This phenomenon is extended in the microbiota of arthropods, with the presence of conserved functional groups, belonging to different classes of proteobacteria, with redundant metabolic capacities [39]. Microorganisms do not exist in isolation but form complex ecological interaction webs, with a positive, negative or no impact on the species involved [40,41]. A particularly important feature of any species is its ability to alter the abundance of other species and shape the whole community, which is referred as a species keystoneness [42]. Network models provide tools for understanding how the microbiota structure can influence host health [41,43,44,45]. Particularly, networks enable the simplified representation of microbial communities as nodes, representing interacting taxa, and links indicating co-occurrence [41]. Microbes that frequently co-occur with many others are referred to as keystone taxa and they play an important role in regulating the microbial community dynamics and functioning [9,46]. Centrality metrics are useful to assess the keystoneness of the nodes, although the centrality metrics best suited to assess keystoneness (i.e., closeness centrality and between centrality) are currently debated [7,46]. The eigenvector centrality (eigencentrality) was proposed as an alternative to closeness centrality and between centrality, since it represents the weighted sum of the centralities of all nodes that are connected to a given node [34]. A high eigenvector score indicates how well a node is connected to other well-connected nodes in the network [47]. In this study, we used three criteria, (i) eigencentrality, (ii) ubiquitousness, and (iii) the combination of relative abundance and eigencentrality, to identify keystone taxa within *Ixodes* microbiota. Despite the differences in the origin of *Ixodes* spp. used for analysis, bacteria of the family Enterobacteriaceae and Corynebacteriaceae were identified as keystone taxa of tick microbiota. Members of the family Enterobacteriaceae have been identified as keystone bacteria in vertebrate [48,49] and plant [50,51] microbiota.

Immunization of C57BL/6 mice with *E. coli* caused increased engorgement weight in *I. ricinus* nymphs. This suggests that *E. coli*, or other Enterobacteriaceae bacteria, may regulate tick feeding or that some metabolites produced by these bacteria may be negative regulators of tick feeding. Enterobacteriaceae may also influence the tick microbial community by ecological cooperation with beneficial bacteria and/or competition with deleterious bacteria. In agreement with this idea, the network analysis revealed positive and negative relationships between bacteria of Enterobacteriaceae and other bacteria present in tick microbiota. Interestingly, a previous study reported that *I. scapularis* larvae that engorged on gentamicin-treated C3H/HeJ mice demonstrated significantly increased engorgement weights, and decreased *B. burgdorferi* colonization when compared to larvae that fed on buffer-treated mice [52]. Gentamicin is active against a wide range of bacterial species, including Gram-negative bacteria of the family Enterobacteriaceae, and the Gram-positive *Staphylococcus*. Altogether, these observations suggest that decreasing the abundance of *E. coli* by immune targeting or antibiotic treatment influences tick feeding by an as yet unknown mechanism. However, it is noteworthy that no changes in the abundance of Enterobacteriaceae was observed in engorged *I. ricinus* nymphs that fed on *E. coli*-immunized C57BL/6 mice.

The fact that ticks that fed on *E. coli*-immunized BALB/c mice did not show an increased engorgement weight as C57BL/6, suggests that host genetic factors or differences in host microbiota may influence the effect of the anti-tick microbiota vaccination based on *E. coli*. The two non-transgenic mice strains used in our study, C57BL/6 and BALB/c, exhibited great differences in gut microbiota [53], and differences in gut microbiota in genetically identical mice explained diverse susceptibilities to vector-borne infections such as malaria [54]. Alternatively, the lack of anti-*E. coli* IgM response in BALB/c may have limited the impact of the vaccination on Enterobacteriaceae and/or the ticks.

Immunization with *E. coli* caused high mortality in *I. ricinus* nymphs that fed on α1,3GT KO, but not in the ticks fed on C57BL/6 or BALB/c mice. Of the mouse strains used in this study, only α1,3GT KO developed both anti-*E. coli* and anti-α-Gal IgM and IgG in response to *E. coli* immunization, while C57BL/6 and BALB/c failed to produce high levels of IgG to α-Gal (C57BL/6) or IgM to *E. coli* and α-Gal (BALB/c). In addition, a significant reduction in the abundance of Enterobacteriaceae was observed only in *E. coli*-immunized α1,3GT KO. Host IgM and IgG induced by *E. coli* immunization may recognize and bind the *E. coli* within the tick microbiota and kill the bacteria. Outer surface protein (OspA) antibodies were shown to kill *Borrelia burgdorferi* spirochetes within feeding nymphs and block transmission to mice in a complement−independent manner [55]. A similar mechanism may account for *E. coli* killing by specific antibodies. The results suggest that in addition to antibodies to *E. coli* proteins, anti-α-Gal immunity may protect against tick infestation.

In this study, however, we could not unravel whether the anti-α-Gal antibodies could also target tick proteins carrying α-Gal. Since the proposal that tick α-Gal was the molecular trigger of the α-Gal syndrome [56], the detection of α-Gal in ticks has been a focus of research. Previous studies reported the presence of α-Gal in *I. ricinus* [57] and *I. scapularis* [58] midguts and salivary gland proteins of *Haemaphysalis longicornis* [59], *Amblyomma sculptum* [60] and *Amblyomma americanum* [58]. Tick proteins with the α-Gal modification were also detected and characterized in salivary gland extracts of *Rhipicephalus bursa, Rhipicephalus microplus* and *Hyalomma marginatum* [30,61]. The enzymes required for the early and late steps of the N-glycosylation pathway [31] and α-Gal synthesis [19] were identified in ticks, suggesting that these arthropods produce endogenous α-Gal. The synthesis of α-Gal by ticks and the presence of this modification in tick salivary proteins may be a mechanism of molecular mimicry to escape the immune response of vertebrate hosts producing endogenous α-Gal and incapable of producing natural anti-α-Gal antibodies [19,31]. The introduction of tick saliva with α-Gal in the human skin through tick bites may trigger a strong immune response to this glycan resulting in allergic reactions and rupture of oral tolerance to this carbohydrate [28]. As previously proposed [62], the ability to produce anti-α-Gal antibodies could make the human a nonsusceptible host to tick infestations.

In this study, we provided for the first time evidence of the genetic basis of α-Gal production by tick microbiota bacteria. Overall, six different α1,3-galactosyltransferase genes were predicted to be present in several bacterial families of the tick gut microbiome, which suggests that tick microbiota could be considered as an additional source of α-Gal in ticks. This finding was confirmed by PCR and sequencing. Enterobacteriaceae, and other bacterial families express α1,3-galactosyltransferase genes and synthetize α-Gal in the human gut microbiome [24]. The production of α-Gal by tick gut microbiota raises the possibility that bacterial molecules containing α-Gal may be introduced to the host through the tick saliva triggering allergic reactions in humans or immune escape in noncatarrhine mammalian hosts.

## 4. Conclusions

In this study, we showed that keystone bacteria of the tick gut microbiota play important roles in tick physiology during feeding and that α1,3-galactosyltransferase genes are widely distributed in the microbiome of *I. scapularis* and *I. ricinus*. Targeting specific vector microbiota bacteria with host antibodies appears to be a suitable tool for the experimental manipulation of the vector microbiota, with implications for studies on vector physiology, control and potentially vector competence for pathogen transmission. Vaccination with *E. coli* induced an antibody response against bacterial proteins and α-Gal. The presence of high levels of anti-*E. coli* and anti-α-Gal IgM and IgG seems to be necessary to induce tick mortality. This antibody response correlated with reduction of tick infestations, likely affecting tick fitness due to a direct effect on commensal microbiota bacteria with α-Gal and to direct action against tick proteins with this modification. The results of this study warrant further experiments to characterize the role of anti-α-Gal immunity in protection against tick infestation and pathogen transmission. The approach described in this study opens up the possibility of using live-bacteria vaccination to target keystone bacteria and study the function of specific members of gut microbiota.

## 5. Materials and Methods

### 5.1. Screenings of Central Bacteria in Tick Gut Microbiota of I. scapularis and I. ricinus Nymphs

#### 5.1.1. Original 16S Data Sets

In the first step of the study, we used published 16S datasets. The original studies described the taxonomic composition of the gut microbiomes of nymphs of *I. scapularis* and *I. ricinus* ticks grown in different conditions in the USA and Europe, respectively (detailed below). These datasets were generated by 251-base paired-end reads from amplicon sequencing of the V4 variable region of the bacterial 16S gene, using barcoded universal primers (515F/806R), sequenced on an Illumina MiSeq system.

(i) The *I. scapularis* data set was described by Abraham et al. [32]. They studied the changes in gut microbiota composition and biofilms of nymphs of *I. scapularis* fed on *A. phagocytophilum*-infected or not infected C3H/HeJ mice housed in laboratory conditions at Yale University, USA. From this data set we used only the samples from nymph fed on uninfected mice (*n* = 10).
(ii) The *I. ricinus* 16S data set was described by Aivelo et al. [33]. They collected free-living ticks from different life stages at three locations in the Swiss Alps, Kanton Graubünden, Switzerland, encompassing a gradient of three heights above sea level. From this data set we only considered the samples representing nymphs (*n* = 10).

#### 5.1.2. Processing of Original Raw Sequences

The raw sequences were downloaded from SRA repository [63], extracted, and de-interlaced in two fastq datasets containing the mate read [64] using the data analysis platform Galaxy (http://usegalaxy.org). The sequences were analyzed using QIIME 2 pipeline (v. 2019.1) [65]. The fastq files were denoised and merged using DADA2 software [66] implemented in QIIME 2. The amplicon sequence variants (ASVs) were aligned with MAFFT [67] (via q2-alignment) and used to construct a phylogeny with FastTree 2 [68] (via q2-phylogeny). Taxonomy was assigned to ASVs using a classify-sklearn naïve Bayes taxonomic classifier [69] based on SILVA database (release 132) [70], only the target sequences fragment was used in the classifier (i.e., classifier trained with the primers) [71,72]. The ubiquitousness of the bacterial families was addressed in each data set by identifying the taxa that persisted across serial fractions of the samples, performed with the QIIME 2 plugin feature-table (core-features) [65].

#### 5.1.3. Bacterial Co-Occurrence Networks

Co-occurrence networks were constructed for each dataset, based on taxonomic profiles, collapsed at family level. The SparCC method [73] implemented in the R environment was used to calculate the correlations matrix. The topological parameter of the networks (i.e., number of nodes and edges, weighted degree, diameter of the network, modularity, and clustering coefficient) were calculated in each data set. Furthermore, we explored the centrality metrics (i.e., betweenness centrality, harmonic centrality, and closeness centrality) [34] for measure the keystoneness of each node, and selected eigenvector centrality for the analysis because it takes into account both the number of connections of a given node and its relevance in terms of influence within the network [47]. Calculations and network visualizations were done with the software Gephi 0.9.2 [74].

#### 5.1.4. Prediction of Functional Traits in Tick Microbiome

The 16S amplicon sequences from each data set were used to predict the metabolic profiling of each sample. PICRUSt2 [36] was used to predict the metagenomes from 16S amplicon sequences. Briefly, the AVSs were placed into a reference tree (NSTI cut-off value of 2) contained 20,000 full 16S sequences from prokaryotic genomes, which is then used to predict individual gene family copy numbers for each AVS. The predictions are based on Kyoto Encyclopedia of Genes and Genomes (KEGG) orthologs (KO) [75].

#### 5.1.5. Identification of α-1,3-Galactosyltransferase Genes in Tick Microbiota Bacteria

The presence of the selected α-1,3-galactosyltransferase genes in bacteria residing tick midguts was confirmed by conventional PCR and sequencing. Prior to tick dissection and genomic DNA extraction, 25 *I. ricinus* female ticks obtained from a laboratory colony (Institute of Parasitology, University of Veterinary Medicine Vienna, Austria) were surface-sterilized with successive washes with 3% hydrogen peroxide, 70% ethanol, and double-distilled water to remove environmental contaminants. Tick midguts were dissected by sterile blades, pooled (five ticks per pool), homogenized, and DNA was extracted using the peqGOLD TriFast DNA extraction procedure (Peqlab, Erlangen, Germany). The bacterial α-1,3-galactosyltransferase genes were amplified using designed primers based on nucleotide sequences available in the KEGG database (K02450, K02847, K03275, K03276, and K03279). DNA extracted from cat feces served as positive control in each PCR run. The primer sequences and PCR amplification conditions are shown in Table 2. Generated PCR products were separated by electrophoresis in 2% agarose gels stained with Midori Green Advance DNA stain (Nippon Genetics Europe, Germany). All positive samples were purified and sequenced in both directions by a commercial company (LGC Genomics, Berlin, Germany). Obtained nucleotide sequences were edited using the software BioEdit (www.mbio.ncsu.edu/BioEdit/bioedit.html) and compared for similarity to sequences available in GenomeNet using BLASTN analysis (https://www.genome.jp/tools/blast/).

### 5.2. Experimental Procedures

#### 5.2.1. Ethics Statement

All procedures in this work were performed according to the principles established by the French and International Guiding Principles for Biomedical Research Involving Animals (2012) at the French Agency for Food, Environmental and Occupational Health & Safety (ANSES) accredited animal facilities in Maisons-Alfort, France. Ethics Committee for Animal Experiments approbation: ComEth Anses/ENVA/UPEC, Permit Numbers E 94 046 08.

#### 5.2.2. Mice and Housing Conditions

BALB/cByJ (Charles River strain code 028) and C57BL/6 (Charles River strain code 027) mice were purchased from Charles River (Miserey, France). The *ggta1* gene knockout mice (α1,3GT KO mice) were bred in a colony maintained at the French Agency for Food, Environmental and Occupational Health & Safety (ANSES) accredited animal facilities in Maisons-Alfort, France. The breeding pairs of α1,3GT KO mice were kindly donated by Dr. Florian Wölbing (Department of Dermatology, Technical University of Munich, Germany). α1,3GT KO mice do not synthesize α-Gal and produce natural anti-α-Gal Abs in titers similar to those in humans, and have been successfully used to test the protective capacity of these antibodies in models of malaria and leishmaniosis [21,76,77]. Six-week-old α1,3GT KO mice from a colony maintained at the French Agency for Food, Environmental and Occupational Health & Safety (ANSES) accredited animal facilities in Maisons-Alfort, France, were used. The animals were maintained in Green line ventilated racks (Tecniplast, Hohenpeissenberg, Germany) at −20 Pa,) with food (Kliba nafaj, Rinaustrasse, Switzerland) and water offered ad libitum. The photoperiod cycles (12 h per day) and room temperature (20–23 °C) were controlled. The animals were monitored twice a day by an experienced technician for any abnormal skin reactions, health problems or complications.

## 6. Bacteria Culture and Life Bacteria Immunization

The bacteria *E. coli* BL21 (DE3, Invitrogen, Carlsbad, CA, USA) were grown on 50 mL of Luria Broth (Sigma-Aldrich, St. Louis, MO, USA), at 37 °C to OD_600_ = 0.8. The bacteria were washed twice with phosphate buffer saline (PBS): 10 mM NaH_2_PO_4_, 2.68 mM KCl, 140 mM NaCl, pH 7.2 (Thermo Scientific, Waltham, MA, USA), centrifuged at 1000× *g* for 5 min at 4 °C, resuspended in PBS at 3.6 × 10^4^ colony-forming unit (CFU)/mL and homogenized with 20 strokes using a glass homogenizer. All reagents used for bacterial preparation were apyrogenic. Six-week-old α1,3GT KO (*n* = 6), C57BL/6 (*n* = 5) and BALB/c (*n* = 5) mice were immunized subcutaneously with *E. coli* in 100 µL (1 × 10^6^ CFU) of a water-in-oil emulsion containing 70% Montanide ISA71 VG adjuvant (SEPPIC, Castres, France), with a booster dose on day 14, two weeks after the first dose. Control α1,3GT KO (*n* = 6), C57BL/6 (*n* = 5), BALB/c (*n* = 5) mice received a mock vaccine containing PBS and adjuvant.

## 7. Sera Collection and Preparation

Blood samples were collected on days 0, 14, 30 and 46 on sterile tubes without anticoagulant. For serum separation, the blood samples were incubated for 2 h (h) at room temperature (RT), allowing for clotting, and then centrifuged at 5000× *g* for 5 min at RT, twice.

## 8. Tick Infestation

Twenty-six days after the second immunization, each mouse was infested with five (α1,3GT KO and BALB/**c**) and twenty (C57BL/6) *I. ricinus* nymphs. The ticks were obtained from the colonies of UMR-BIPAR, Maisons-Alfort, France. Ticks were fed on experimental mice within the EVA-foam (Cosplay Shop, Brugge, Belgium) capsule glued to the animal backs [78]. Engorged nymphs were kept in a polypropylene tube and maintained with a light-dark (12 h/12 h) cycle in a glass desiccator with >97% relative humidity at 22 °C.

## 9. Protein Extraction

The *E. coli* was washed twice with PBS, centrifuged at 1000× *g* for 5 min at 4 °C, resuspended in 7 M Urea lysis buffer (Sigma-Aldrich, St. Louis, MO, USA) and homogenized with 20 strokes using a glass balls homogenizer. The homogenate was centrifuged at 300× *g* for 5 min at 4 °C and the supernatant was collected. Protein concentration was determined using the Bradford Protein Assay (Thermo Scientific, San Jose, CA, USA) with Bovine Serum Albumin (BSA) as standard.

## 10. Indirect ELISA for Anti-α-Gal and Anti-BL21 Protein Antibodies

To evaluate the levels of specific Abs against Galα1-3Gal (α-Gal), and *E. coli* protein extract as protocols previously published were used [15,16,17,23], with slight modifications. The 96-well ELISA plates (Thermo Scientific, Waltham, MA, USA) were coated with 0.5 µg/mL (100 µL/well) of either Galα1-3Gal linked to BSA (Dextra Laboratories, Reading, UK) or *E. coli* protein extract and incubated for 2 h with 100 rpm shaking at room temperature. Subsequently, plates were incubated overnight at 4 °C. The antigens were diluted in carbonate/bicarbonate buffer (0.05 M, pH 9.6) and incubated overnight at 4 °C. Optimal antigen concentration and dilutions of sera and conjugate were defined using a titration assay. Wells were washed three times with 100 µL of PBS containing 0.05% (vol/vol) Tween 20 (PBST), and then blocked by adding 100 µL of 1% Human Serum Albumin (HSA)/PBS for 1 h at RT and 100 rpm shaking. After three washes, serum samples, diluted in 0.5% HSA/PBS (1:50 or 1:500 for BALB/c mice), were added to the wells and incubated for 1 h at 37 °C with shaking. The plates were washed three times and HRP-conjugated Abs (goat anti-mice IgG and IgM) (Sigma-Aldrich, St. Louis, MO, USA) were added at 1:1500 dilution in 0.5% HSA/PBST (100 µL/well) and incubated for 1 h at RT with shaking. The plates were washed three times and the reaction was developed with 100 µL ready-to-use TMB solution (Promega, Madison, WI, USA) at RT for 20 min in the dark, and then stopped with 50 µL of 0.5 M H2SO4. The OD values were measured at 450 nm using an ELISA plate reader (Filter-Max F5, Molecular Devices, San Jose, CA, USA). All samples were tested in triplicate and the average value of three blanks (no Abs) was subtracted from the reads. The cut-off was determined as two times a mean OD value of the blank controls.

## 11. Relative Quantification of Enterobacteriaceae by qPCR

### 11.1. DNA Extraction

DNA was extracted from nymphs that fed on C57BL/6 and the nymphs that fed on α1,3GT KO and BALB/c were allowed to molt to adults. Nymphs and adults were pooled (*n* = 5 ticks per pool) and crushed with glass beads using a Precellys24 Dual homogeniser (Bertin technologies, Paris, France) at 5500× rpm for 20 s. Genomic DNA was then extracted using a Nucleospin tissue DNA extraction Kit (Macherey-Nagel, Hoerdt, France). For each sample, total DNA was eluted in 75 µl of sterile water and stored at −20 °C until further analysis.

### 11.2. qPCR

To quantify the relative amount of Enterobacteriaceae bacteria, a qPCR was carried out using primers and protocol previously reported [79]. A 16S rRNA gene amplification was performed with SYBR Green LightCycler 480 Master mix (Roche, Meylan, France) and the conditions as follow: 50 °C for 2 min, 95 °C for 10 min, 40 cycles of 95 °C for 15 s and 60 °C for 1 min. The CT values were recorded, and the relative levels of bacterial DNA were normalized against tick *rsp4* as housekeeping gene. Fold change in relative quantities were then calculated using the 2^−ΔΔ*C*t^ ratio method [80].

## 12. Statistical Analysis

Differences in antibody levels (OD) between groups in the different time points were evaluated using two-way ANOVA with Bonferroni multiple comparison test applied for individual comparisons. Unpaired non-parametric Mann−Whitney U test was used to compare the tick parameters and Enterobacteriaceae levels between groups. All analyses were performed in the GraphPad 5 Prism program (GraphPad Software Inc., San Diego, CA, USA). Differences were considered significant when *p* < 0.05.

## Figures and Tables

**Table 1:** Eigencentrality and relative abundance values of ubiquitous bacterial families in *Ixodes* spp. ticks.

Ubiquitous Bacterial Families	Eigencentrality *		Relative Abundance *	
	*I. scapularis*	*I. ricinus*	*I. scapularis*	*I. ricinus*
Enterobacteriaceae	0.69	0.69	17.83	5.36
Corynebacteriaceae	0.79	0.85	5.76	2.04
Pseudomonadaceae	0.42	0.67	5.66	19.42
Sphingomonadaceae	0.71	0.40	0.96	9.61

* Values as in Supplementary Table S1.

**Table 2:** Primers and PCR conditions used for amplification of α-1,3-galactosyltransferase genes from midgut bacteria of *Ixodes ricinus* ticks.

Gene Names (Reference Accessions)	Primer Sequences (5′–3′)	Annealing Temperature (°C)	Fragment Length (bp)
*gspA* (K02450)	1. reaction gspA_F: GTTGGGTGAGGCTGGAAGTG gspA_R: TGCGATCCAGGGCAAATTCTG	60	958
	2. reaction (nested) gspAnest_F: TGAATCACCGCCGCATACT gspAnest_R: CTCATCACCACGGCAAGCT	58	406
*waaL*, *rfaL* (K02847)	waaL_F: CAATGCATATCGTGGCCCAA waaL_R: CCGACTGAAGTTCTGGTGTT	58	573
*waaO*, *rfaI* (K03275)	waaO_F: GAACGGCTTAAGGCATTACC waaO_R: CTTGGCGCAATAACGAGCA	56	654
*waaR*, *waaT* and *rfaJ* (K03276)	waaR_F: GTACACAGGTCTGGTCAAGA waaR_R: CAGACTCCTGCTATAATTCCTG	56	685
*waaJ* and *rfaJ* (K03279)	waaJ_F: CCGTTAAACTTGATGAACGGGAA waaJ_R: TAGTTCGCCCAATCATGCCA	60	748

## Supplementary Materials

The following are available online at https://www.mdpi.com/2076-393X/8/4/702/s1, Table S1. Values of eigencentrality and relative abundance of bacteria taxa in *I. scapularis* and *I. ricinus* microbiota.
