# Supplementary material for: Anti-Tick Microbiota Vaccine Impacts Ixodes ricinus Performance during Feeding
Source: Vaccines (Basel). 2020 Nov 21;8(4):702. doi: 10.3390/vaccines8040702 (PMC7711837; doi:10.3390/vaccines8040702)
Supplement: Supplementary file 1 [file vaccines-08-00702-s001.zip › vaccines-996254-supplementary.pdf]

Family	Relative abundance (%)	Eigencentrality
Aeromonadaceae	0.23	#N/A
Streptococcaceae	0.23	#N/A
Lactobacillales	0.06	#N/A
Intrasporangiaceae	0.04	#N/A
Saprospiraceae	0.04	#N/A
Listeriaceae	0.03	#N/A
env.OPS 17	0.03	#N/A
Sphingobacteriaceae	0.03	#N/A
Frankiaceae	0.02	#N/A
Brevibacteriaceae	0.02	#N/A
Veillonellaceae	0.02	#N/A
Mycobacteriaceae	0.02	#N/A
Blastocatellaceae	0.02	#N/A
Verrucomicrobiaceae	0.02	#N/A
Bdellovibrionaceae	0.02	#N/A
Hyphomicrobiaceae	0.01	#N/A
Gaiellales	0.01	#N/A
Gammaproteobacteria	0.01	#N/A
Solibacteraceae (Subgroup 3)	0.01	#N/A
Porphyromonadaceae	0.01	#N/A
Flavobacteriaceae	0.01	#N/A
Kineosporiaceae	0.01	#N/A
Cellulomonadaceae	0.01	#N/A
Acidobacteriaceae (Subgroup 1)	0.01	#N/A
Family XIII	0.01	#N/A
Christensenellaceae	0.01	#N/A
Opitutaceae	0.01	#N/A
67-14	0.01	#N/A
Micrococcales	0.01	#N/A
Rhodopirillaceae	0.01	#N/A
Dermabacteraceae	0.01	#N/A
Vibrionaceae	0.01	#N/A
Acido.Subgroup 6	0.01	#N/A
A4b	0.01	#N/A
Cytophagaceae	0.01	#N/A
Arcobacteraceae	0.01	#N/A
Chroococcidiopsaceae	0.01	#N/A
Family X	0.01	#N/A
Geodermatophilaceae	0.01	#N/A
Hydrogenophilaceae	0.01	#N/A
Solimonadaceae	0.01	#N/A
KD4-96	0.01	#N/A
SC-I-84	0.01	#N/A
Muribaculaceae	0.01	#N/A
Bacteroidales	0.01	#N/A
uncultured Ferrimicrobium sp.	0.00	#N/A
Pedosphaeraceae	0.00	#N/A
SJA-28	0.00	#N/A
uncultured Chlorobiales bacterium	0.00	#N/A

Family XII	0.00	#N/A
Mycoplasmataceae	0.00	#N/A
Nakamurellaceae	0.00	#N/A
Paracaedibacteraceae	0.00	#N/A
Reyranellaceae	0.00	#N/A
Acidaminococcaceae	0.00	#N/A
Steroidobacteraceae	0.00	#N/A
WD2101 soil group	0.00	#N/A
Gemmataceae	0.00	#N/A
Haliangiaceae	0.00	#N/A
Phormidiaceae	0.00	#N/A
Rhodocyclaceae	0.00	#N/A
Pla1 lineage	0.00	#N/A
Microscillaceae	0.00	#N/A
Betaproteobacteriales	0.00	#N/A
Sporichthyaceae	0.00	#N/A
Marinifilaceae	0.00	#N/A
Acidimicrobiia	0.00	#N/A
Atopobiaceae	0.00	#N/A
Nocardiopsaceae	0.00	#N/A
Frankiales	0.00	#N/A
Micromonosporaceae	0.00	#N/A
Microtrichales	0.00	#N/A
ncultured Termite group 1 bacteriu	0.00	#N/A
Streptomycetaceae	0.00	#N/A
Gaiellaceae	0.00	#N/A
Paludibacteraceae	0.00	#N/A
Chitinophagales	0.00	#N/A
Devosiaceae	0.00	#N/A
Kaistiaceae	0.00	#N/A
Alphaproteobacteria	0.00	#N/A
Archangiaceae	0.00	#N/A
Ectothiorhodospiraceae	0.00	#N/A
Planococcaceae	25.09	1.00
Bifidobacteriaceae	0.08	0.97
Staphylococcaceae	3.60	0.88
Bacillaceae	0.28	0.86
Bogoriellaceae	0.04	0.82
Caulobacteraceae	0.24	0.80
Enterococcaceae	1.32	0.79
Corynebacteriaceae	5.76	0.79
Methylophilaceae	0.03	0.77
Burkholderiaceae	6.28	0.73
Erysipelotrichaceae	0.05	0.72
Solirubrobacteraceae	0.05	0.72
Anaplasmataceae	0.62	0.72
Paenibacillaceae	0.08	0.71
Sphingomonadaceae	0.96	0.71
Micrococcaceae	0.29	0.69
Enterobacteriaceae	17.83	0.69

Halomonadaceae	0.07	0.69
Dermacoccaceae	0.64	0.69
Nocardioidaceae	0.03	0.68
Microbacteriaceae	0.11	0.67
Rhodanobacteraceae	0.20	0.67
Aerococcaceae	0.04	0.67
Moraxellaceae	15.86	0.65
Xanthomonadaceae	1.77	0.64
Propionibacteriaceae	0.03	0.62
Myxococcales	0.02	0.60
Nocardiaceae	0.06	0.59
Hymenobacteraceae	0.02	0.58
Carnobacteriaceae	0.14	0.57
Pseudonocardiaceae	0.03	0.57
Rhizobiaceae	0.44	0.57
Xanthobacteraceae	0.09	0.57
Lactobacillaceae	0.08	0.54
Rickettsiales	0.16	0.53
Oligoflexaceae	0.23	0.53
Clostridiaceae 1	0.07	0.53
Actinomycetaceae	0.10	0.52
Rhodobacteraceae	0.09	0.50
Beijerinckiaceae	0.26	0.49
Pirellulaceae	0.00	0.49
Acetobacteraceae	0.23	0.49
Neisseriaceae	1.62	0.48
Lachnospiraceae	0.30	0.47
Bacillales	0.05	0.46
Gastranaerophilales	0.00	0.46
Bacteroidaceae	0.08	0.45
Thermaceae	0.10	0.43
Alteromonadaceae	0.01	0.43
Prevotellaceae	0.19	0.42
Pseudomonadaceae	5.66	0.42
Peptococcaceae	0.00	0.40
Rickettsiaceae	5.14	0.40
Pleomorphomonadaceae	0.14	0.40
Pasteurellaceae	0.05	0.39
Succinivibrionaceae	0.02	0.39
Rikenellaceae	0.05	0.39
Mollicutes RF39	0.00	0.38
Fusobacteriaceae	0.06	0.38
iammaproteobacteria Incertae Sed	0.02	0.37
Leptotrichiaceae	0.00	0.36
Desulfovibrionaceae	0.00	0.36
0319-6G20	0.02	0.34
Spirosomaceae	0.01	0.33
Gemmatimonadaceae	0.03	0.32
Weeksellaceae	0.16	0.32
Peptostreptococcaceae	0.01	0.31

Clostridiales vadinBB60 group	0.01	0.31
Legionellaceae	0.01	0.30
Ruminococcaceae	0.16	0.29
Deinococcaceae	0.01	0.29
Anaerolineaceae	0.00	0.29
Proteobacteria	0.00	0.28
Clostridiaceae 2	0.01	0.27
Campylobacteraceae	0.07	0.26
Polyangiaceae	0.00	0.26
Shewanellaceae	0.00	0.26
R7C24	0.00	0.25
A0839	0.00	0.25
Parachlamydiaceae	0.01	0.25
Chitinophagaceae	0.02	0.25
Spirochaetaceae	0.01	0.24
Nitrospiraceae	0.00	0.24
Family XI	1.31	0.23
Family XI	0.01	0.23
Dietziaceae	0.00	0.23
Tannerellaceae	0.00	0.22
JG30-KF-CM45	0.01	0.22
WD260	0.02	0.22
Dysgonomonadaceae	0.00	0.21
Cellvibrionaceae	0.00	0.20
Leuconostocaceae	0.01	0.19
