# Supplementary material for: Anti-Tick Microbiota Vaccine Impacts Ixodes ricinus Performance during Feeding
Source: Vaccines (Basel). 2020 Nov 21;8(4):702. doi: 10.3390/vaccines8040702 (PMC7711837; doi:10.3390/vaccines8040702)
Supplement: Supplementary file 1 [file vaccines-08-00702-s001.zip › vaccines-996254-supplementary2.pdf]

Familiy	Relative abundance (%)	Eigencentrality
Pseudomonadaceae	19.42	0.67
Midichloriaceae	17.05	1.00
Staphylococcaceae	10.80	0.14
Sphingomonadaceae	9.61	0.40
Enterobacteriaceae	5.36	0.69
Planococcaceae	4.98	0.80
Brevibacteriaceae	4.03	0.32
Moraxellaceae	3.94	0.63
Burkholderiaceae	3.63	0.12
Sphingobacteriaceae	2.31	0.63
Wohlfahrtiimonadaceae	2.14	0.83
Corynebacteriaceae	2.04	0.85
Micrococcaceae	1.87	0.19
Chitinophagaceae	1.59	0.13
Flavobacteriaceae	1.58	0.33
Dermabacteraceae	1.48	0.57
Mitochondria	1.48	0.39
Xanthomonadaceae	1.22	0.61
Diplorickettsiaceae	0.95	0.65
Propionibacteriaceae	0.78	0.71
Dietziaceae	0.60	0.19
Hymenobacteraceae	0.55	0.29
Beijerinckiaceae	0.44	0.70
Rhizobiaceae	0.43	0.63
Neisseriaceae	0.36	0.82
Enterococcaceae	0.35	0.42
Nocardiaceae	0.26	0.70
Caulobacteraceae	0.23	0.19
Rhodobacteraceae	0.23	0.16
Microbacteriaceae	0.20	0.36
Lactobacillaceae	0.07	0.26
